# A novel function of the human oncogene Stil: Regulation of PC12 cell toxic susceptibility through the Shh pathway

**DOI:** 10.1038/srep16513

**Published:** 2015-11-09

**Authors:** Lei Li, Aprell L. Carr, Lei Sun, Audrey Drewing, Jessica Lee, Zihe Rao

**Affiliations:** 1 Department of Biological Sciences, University of Notre Dame, Notre Dame, IN 46556; 2 Tianjin International Joint Academy of Biotechnology and Medicine, Tianjin, China 300457

## Abstract

The human oncogene SCL/TAL1 interrupting locus (Stil) is highly conserved in vertebrate species. Here, we report new findings of Stil in the regulation of toxic susceptibility in mammalian dopaminergic (DA)-like PC12 cells. RNAi-mediated knockdown of Stil expression did not affect the survival of proliferating PC12 cells but caused a significant amount of cell death in differentiated neurons after toxic drug treatment. In contrast, overexpression of Stil increased toxic susceptibility only in proliferating cells but produced no effect in mature neurons. Exogenetic inactivation or activation of the Sonic hedgehog (Shh) signaling transduction mimicked the effect of Stil knockdown or overexpression in regulation of PC12 cell toxic susceptibility, suggesting that Stil exerts its role through the Shh pathway. Together, the data provide evidence for novel functions of the human oncogene Stil in neural toxic susceptibility.

STIL is a ubiquitous-expressed protein in many different cell types and it contains a STAN motif that is similar to the C-terminus of TGF-β^1^,^2^,^3^. STIL is highly conserved in vertebrate species where it functions as a cell-cycle checkpoint protein that regulates the G2/M transition for mitotic entry and spindle pole organization^4^,^5^,^6^,^7^. In mice, deletion of Stil loci results in abnormal development of body axes, and the animals die during late embryonic stages^8^. Previous studies have demonstrated that STIL functions in the Shh pathway, i.e., STIL binds cytoplasmic SUFU protein, which frees GLI1 from SUFU repression for translocation to the nucleus and for target gene transcription^9^,^10^. *In vivo* studies using the zebrafish mutants (*csp*^cz65^, which is a homolog of *Stil*) indicated that STIL is required for spindle pole organization during cell proliferation^11^. In human cancer cells, STIL localizes to the pericentriolar region in centrosomes during metaphase. This is essential for its additional roles in spindle pole positioning as well as centriole formation and duplication^12^,^13^,^14^.

Recent studies have shown that Stil also plays important roles in nerve system development and survival. In zebrafish mutants (e.g., *nbb*^*da15*^), for example, down-regulation of Stil expression interrupted cell proliferation and caused dopaminergic (DA) amacrine cell degeneration^15^,^16^. However, such defects can be rescued by up-regulating Shh signaling transduction (e.g., inhibition of SUFU expression)^17^,^18^. In mammalian DA-like PC12 cells, the expression of Stil is required for cell proliferation. For example, shRNA-mediated knockdown of Stil expression decreased the rate of cell proliferation, whereas overexpression of Stil mRNA increased PC12 cell proliferation^19^. Considering the conserved expression of Stil in different species and cell types, the role of Stil in cell proliferation and neural degeneration, and the function of Shh signaling in regulation of drug sensitivity, it is conceivable to hypothesize that Stil plays a role in the regulation of DA cell toxic susceptibility.

In this study, we investigate the role of STIL expression in PC12 cells in response to assaults by neurotoxins. We demonstrate that the effects of Stil on PC12 cell survival and apoptosis are mediated by Shh and Caspase-3 mediated signal transduction pathways. These findings provide evidence of differential effect of Stil expression in proliferating and differentiated cells, and reveal a novel function for Stil in neural protection.

## Results

**Figure 1:**
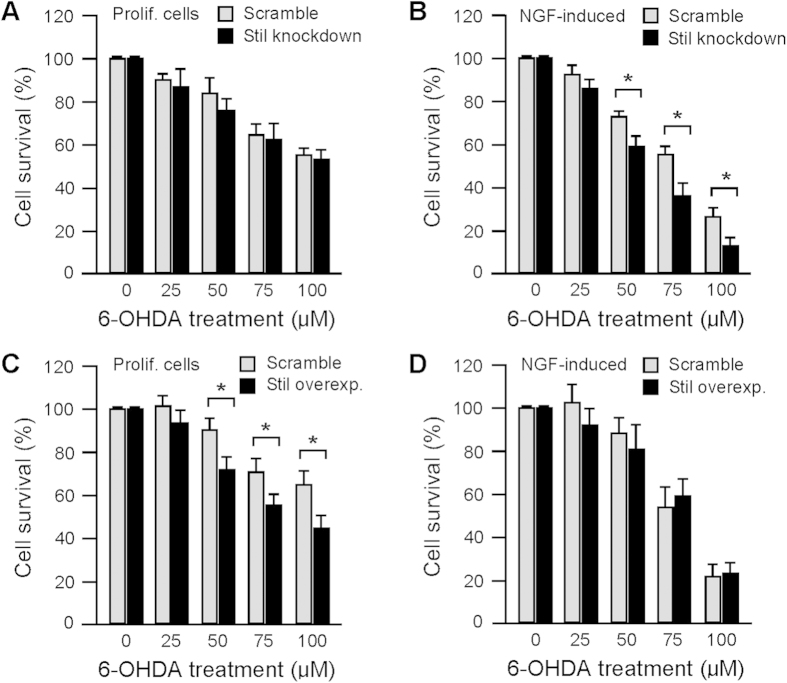
Effects of Stil expression on PC12 cell’s toxic susceptibility. (**A**) In proliferating PC12 cells, both the control and Stil-knockdown cells showed dose-dependent decreases in cell survival in response to 6-OHDA treatment. No significant differences in cell survival were detected between the treatment and control cells. (**B**) In NGF-induced mature cells, knockdown of Stil expression decreased the rate of cell survival after 6-OHDA treatment, especially when treated with high concentrations of 6-OHDA. (**C**) In proliferating cells, overexpression of Stil decreased the rate of cell survival as compared to control cells, especially when tested with high concentrations of 6-OHDA. (**D**) In NGF-induced mature cells, overexpression of Stil produced no effect on cell survival as compared to control cells. Data represent the Means ± SE, n = 6; *p < 0.01.

Mammalian PC12 cells display dose-dependent toxic responses to treatment with neurotoxins, such as 6-hydroxydopamine (6-OHDA)^20^,^21^,^22^. Up- or down-regulation of Stil expression differentially regulates the toxic susceptibility of PC12 cells depending on the status of the cells, i.e., if the cells are under proliferation or already differentiated. In proliferating PC12 cells, knockdown of Stil expression (by transfection with pSIREN-Stil-shRNA plasmid) produced no effect on cell survival in response to 6-OHDA treatment. Under all tested concentrations (6-OHDA applied at 0, 25, 50, 75 and 100 μM), the rate of cell survival was similar in Stil-knockdown cells and control cells (transfected with scramble sequence plasmid) (Fig. 1A). However, in nerve growth factor (NGF)-induced differentiated PC12 cells, knockdown of Stil expression resulted in significant increases in drug toxicity. For example, in response to 50, 75, and 100 μM 6-OHDA treatment, the survival rates of Stil-knockdown cells (60.1 ± 4.8, 36.7 ± 5.2, and 12.8 ± 4.1%) were significantly lower than the survival rates in control cells (73.2 ± 2.7, 55.1 ± 4.6, and 25.8 ± 4.9%) (Fig. 1B).

Overexpression of Stil (by transfection with full-length human Stil sequence using pCS2+Flh-Stil plasmid) increased the toxic susceptibility of PC12 cells but only in proliferating cells. For example, when tested using 50, 75 and 100 μM 6-OHDA, the survival rate of Stil-overexpression proliferating cells was 72.1.1 ± 5.1, 55.8 ± 4.9, and 45.0 ± 5.2%, respectively, whereas in control cells (transfected with scramble sequence plasmid), the survival rate was 89.8 ± 5.3, 70.7 ± 6.2, and 64.7 ± 5.1% (Fig. 1C). In NGF-induced PC12 cells, overexpression of Stil produced no effect on toxic susceptibility and the cell survival rates in all tested concentrations (6-OHDA applied at 0, 25, 50, 75 and 100 μM) were similar in Stil-overexpression cells and control cells (Fig. 1D).

**Figure 2:**
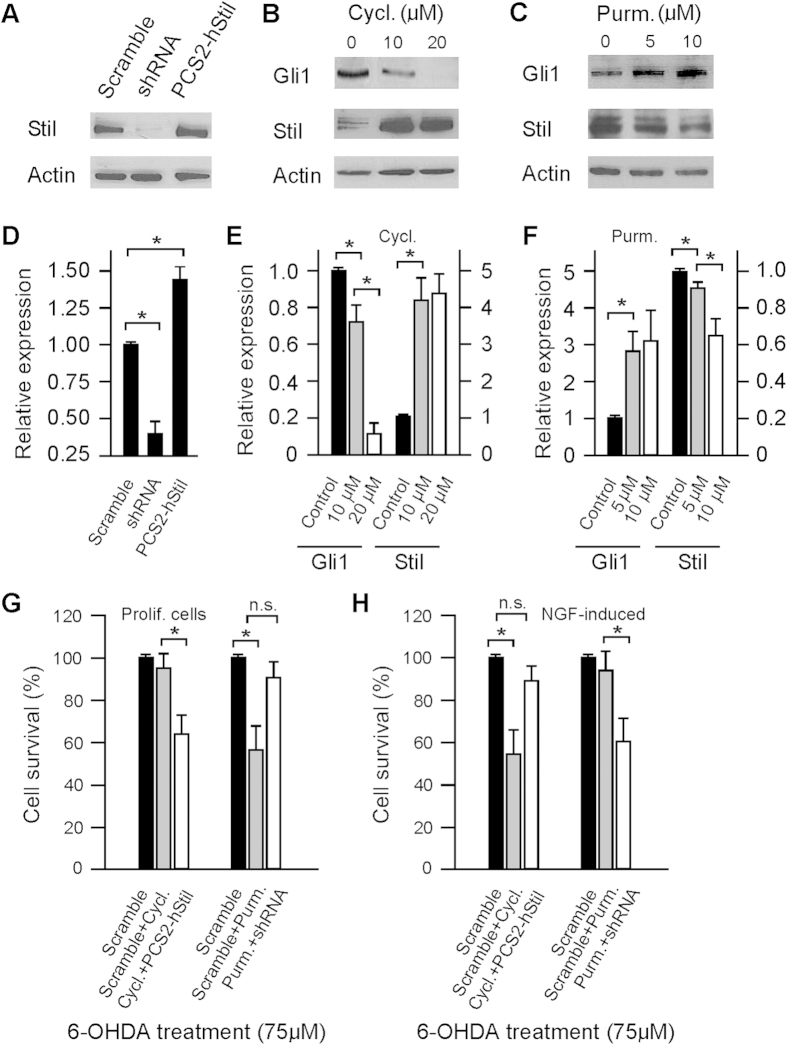
Effects of Shh signaling on Stil expression and PC12 cell survival in response to toxic drug treatment. (**A**) Western blot of STIL proteins in PC12 cells after transfection with pSIREN-Stil-shRNA (knockdown) or pCS2+Flh-Stil plasmid (overexpression). The gels have been run under the same experimental conditions. (**B**,**C**) Western blot of STIL and GLI1 proteins in PC12 cells after treatment with Smo receptor agonist cyclopamine or Smo receptor antagonist purmorphamine. Treatment with cyclopamine decreased GLI1 expression but resulted in the accumulation of STIL proteins. Treatment with purmorphamine promoted Shh signaling but caused a decrease in STIL protein expression. All the gels have been run under the same experimental conditions. (**D**) RT-PCR analyses of Stil mRNA expression in PC12 cells transfected with pSIREN-Stil-shRNA or pCS2+Flh-Stil plasmid. Note the alterations in Stil mRNA expression in response to plasmid transfection. (**E**,**F**) RT-PCR analyses of Stil and Gli1 expression in response to cyclopamine or purmorphamine treatment in PC12 cells. Treatment with either compound resulted in significant changes in the expression of Stil or Gli1 mRNA. (**G**,**H**) Cell survival in response to 6-OHDA treatment (75 μM) in proliferating and NGF-induced mature cells while the Shh signaling transduction was modified by plasmid transfection. Data represent the Means ± SE, n = 4; n.s., not significant, *p < 0.01.

Previous studies have demonstrated that Stil functions in the Shh pathway in DA neurons^9^,^10^. To investigate if the effect of Stil in regulation of PC12 cell toxic susceptibility is mediated by Shh signaling transduction, we measured PC12 cell survival in response to 6-OHDA treatment while the transduction of the Shh signals was modified by knockdown/overexpression of the Stil transcript or by inhibition/activation of Smo receptors which function in the Shh pathway^23^,^24^,^25^. In proliferating PC12 cells, transfection with pSIREN-Stil-shRNA or pCS2+Flh-Stil altered the level of STIL protein expression (Fig. 2A,D). Inhibition or activation of Smo receptors (by treatment with Smo receptor antagonist cyclopamine or receptor agonist purmorphamine) not only altered Shh signaling transduction (i.e., Gli1 transcription), but also modified the expression of STIL proteins. In response to cyclopamine treatment (10 and 20 μM), for example, the expression of GLI1 was decreased to levels that were 71.8 ± 9.8 and 11.0 ± 7.2% of the control cells, and the expression of STIL was increased to levels approximately 4–5 folds higher than controls (Fig. 2B,E). In purmorphamine (5 and 10 μM) treated cells, the expression of Gli1 was increased by 3–4 folds as compared to control cells but the expression STIL proteins was decreased, i.e., to 90.3 ± 3.6 and 65.1 ± 9.5% of the control level (Fig. 2C,F).

In proliferating PC12 cells, inhibition of Shh signaling transduction (i.e., by cyclopamine treatment) produced no obvious effect on cell’s drug susceptibility to 6-OHDA (Fig. 2G). However, when the Shh signaling in cyclopamine-treated cells was factitiously elevated, i.e., by overexpression of Stil transcripts (transfected with pCS2+Flh-Stil plasmid), the toxic susceptibility of PC12 cells to 6-OHDA was increased. For example, in response to 6-OHDA treatment (75 μM), the survival rate of cyclopamine-treated and plasmid-transfected cells was decreased to 64.9 ± 8.7% of the control level (Fig. 2G). Activation of Shh signaling transduction (by treatment with purmorphamine) increased the toxic drug susceptibility of proliferating PC12 cells, but this could be reversed by factitious down-regulation of Shh signaling transduction. In purmorphamine-treated cells, for example, in response to 6-OHDA treatment (75 μM) the survival rate was decreased to 56.8 ± 10.3% of the control level (Fig. 2G). Down-regulation of Stil expression (by transfection with pSIREN-Stil-shRNA plasmid) in purmorphamine-treated cells blocked the effect of Smo receptor-initiated Shh signaling on drug toxicity, i.e., in response to 6-OHDA treatment the survival rate of the cell was increased to the control level (Fig. 2G).

In NGF-induced PC12 cells, inhibition of Shh signaling transduction (by cyclopamine) increased PC12 cell’s toxic susceptibility, thereby decreasing the rate of cell survival (i.e., to 55.0 ± 10.8% of the control level) (Fig. 2H). A complete rescue in cell survival could be achieved by increasing STIL expression (by transfection with pCS2+Flh-Stil plasmid) (Fig. 2H). Activation of the Shh pathway (by purmorphamine) produced no effect on drug susceptibility in differentiated PC12 cells (Fig. 2H). However, when the expression of Stil was factitiously inhibited (transfection with pSIREN-Stil-shRNA plasmid), the survival rate of purmorphamine-treated cells was decreased (i.e., to 60.3 ± 10.2% of the control level) (Fig. 2H). In this case, although the early signaling events in the Shh pathway were promoted, the production of STIL was inhibited by shRNA and thereby the transcription of Gli1 was inhibited.

**Figure 3:**
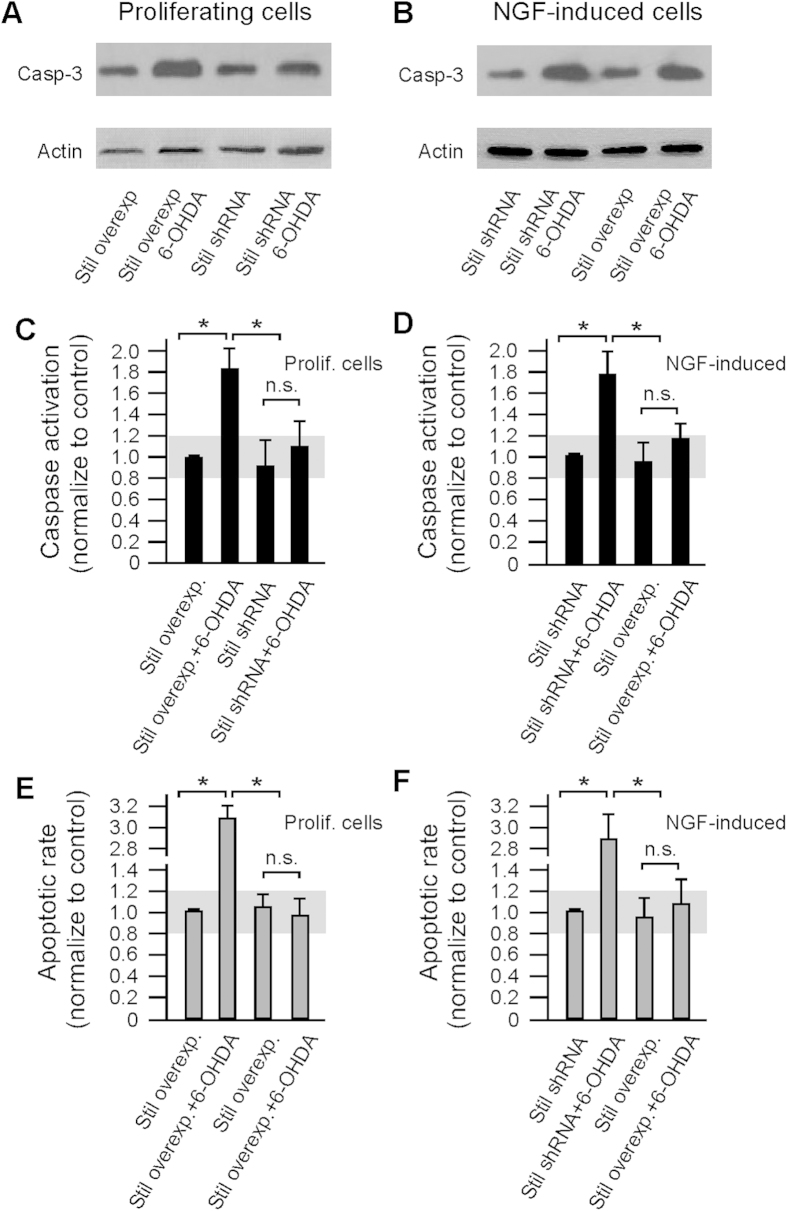
Caspase-3 mediated apoptotic cell death in PC12 cells in response to 6-OHDA treatment (75 μM). (**A**,**B**) Western blot of cleaved Caspase-3 proteins in proliferating and NGF-induced mature cells in response to sham or 6-OHDA treatment. Note the increase in cleaved Caspase-3 expression in proliferating cells after Stil-overexpression and in differentiated cells after Stil-knockdown. The gels have been run under the same experimental conditions. (**C**,**D**) Caspase activity in proliferating and NGF-induced PC12 cells in transfected cells before and after 6-OHDA treatment. Gray bars indicate the range of caspase activity in control cells. Note the increase of Caspase activation in proliferating cells in response to Stil overexpression and in mature cells in response to Stil knockdown after 6-OHDA treatment. (**E**,**F**) TUNEL analyses of apoptotic cell death in PC12 cells. Gray bars indicate the range of cell death in control cells. Note the increase of cell death in proliferating cells in response to Stil overexpression and in mature cells in response to Stil knockdown after 6-OHDA treatment. Data represent the Means ± SE, n = 4; n.s., not significant, *p < 0.01.

The decrease of cell survival of PC12 cells after 6-OHDA treatment is due to the increase of caspase-mediated cell death. This was verified by analyzing cleaved Caspase-3 protein expression, caspase activation, and by using the TUNEL assay. Western blot revealed significant increases in the expression of cleaved Caspase-3 proteins in proliferating PC12 cells in response to 6-OHDA treatment when the expression of Stil was elevated (i.e., in cells transfected with pCS2+Flh-Stil plasmid) (Fig. 3A). Caspase activation assays also revealed an increase (1.8 ± 0.2 folds) of caspase activity in Stil-overexpression cells as compared to control cells in response to 6-OHDA treatment (Fig. 3C). In contrast, in proliferating PC12 cells knockdown of Stil expression (by transfecting the cells with pSIREN-Stil-shRNA plasmid) produced no obvious effects on Caspase-3 expression and caspase activity after 6-OHDA treatment as compared to control cells (transfected with scramble sequence plasmid) (Fig. 3A,C). The increase of caspase activity led to increased apoptotic cell death revealed by the TUNEL assay. For example, in proliferating PC12 cells, in response to 6-OHDA treatment, the amount of cell death in Stil overexpression cells was 3.1 ± 0.1 folds higher than the rate of cell death in control cells (Fig. 3E). In NGF-induced PC12 cells, in response to 6-OHDA treatment increases in cleaved Caspase-3 expression and caspase activation were seen in Stil-knockdown cells, but not in Stil-overexpression cells (Fig. 3B,D). Increases in apoptotic cell death were seen in Stil-knockdown cells when treated with 6-OHDA as revealed by the TUNEL assay (Fig. 3F).

## Discussion

In vertebrates, STIL is expressed in different cell types where it regulates the process of cell proliferation. In a recent study, we examined the role of STIL in neural degeneration and regeneration in zebrafish models and mammalian PC12 cells. In zebrafish retinas, functional expression of STIL is required for DA cell regeneration after drug-induced degeneration^17^,^18^. In mammalian PC12 cells, the expression of STIL is required for cell proliferation but not neural differentiation^19^.

In this research, we examined the function of STIL in PC12 cell survival in response to treatment with neurotoxin 6-OHDA. RNAi-mediated knockdown of Stil mRNA expression efficiently diminished the expression of STIL proteins, whereas overexpression of the full-length human Stil sequence promoted STIL expression^19^. In addition, we manipulated the Shh signaling transduction using pharmacological approaches. Down-regulation of Shh signaling was achieved by treatment with the steroidal alkaloid cyclopamine, which functions as an antagonist of the Smo receptor in the Shh pathway^26^. Up-regulation of Shh signaling was achieved by using a small molecule purmorphamine, which is an agonist for Shh receptor Smo^27^. Treatment with cyclopamine or purmorphamine does not induce growth arrest or trigger cell death in PC12 cells, which thereby provide a tool that allow functional analyses of the effect of STIL knockdown or overexpression in cell survival in the presence or absence of Shh signaling transduction. In proliferating PC12 cells, down-regulation of Stil expression produced no obvious effect on cells’ drug susceptibility. However, increase of Stil expression resulted in increases in drug susceptibility. This is likely mediated by the Shh signaling transduction pathway. In cyclopamine-treated proliferating PC12 cells, for example, the activity of the Shh pathway was decreased. However, the same drug treatment resulted in significant increases of Stil expression (see Fig. 2B). The increase of Stil expression may counter the effect of cyclopamine treatment, and thereby increase the activity of Shh signaling transduction. Transfection of cyclopamine-treated PC12 cells with pCS2+Flh-Stil plasmids increases the expression of Stil mRNA, which in turn, increases Shh transduction, and eventually increases the drug susceptibility of the cell (see Fig. 2G). In differentiated PC12 cells, Stil exerts its role on cell drug susceptibility in opposite ways, that is, overexpression of Stil produced no obvious effect on cells’ survival in response to 6-OHDA treatment, but knockdown of Stil expression increases the rate of cell death after toxic drug insults. In differentiated mature PC12, the effect of Stil on drug susceptibility is also mediated by the Shh signaling transduction pathway.

**Figure 4:**
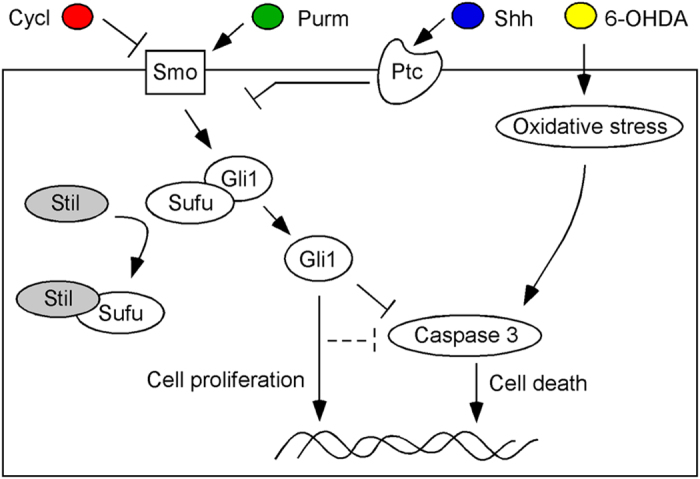
Possible mechanisms of Stil in the Shh pathway in the regulation of PC12 cell toxic susceptibility. Normally, STIL binds SUFU and free GLI1 for downstream gene transcription. Inactivation of Smo receptors by cyclopamine inhibits the Shh pathway, but the downstream Gli1 signaling can be resumed by overexpression of STIL. Activation of Smo receptors by purmorphamine promotes Shh signaling, but the effect of purmorphamine can be diminished by down-regulation of STIL expression. In mature cells, the increase of Gli1 transcription inhibits the Caspase-3 activity and thereby prevents cell apoptosis. In proliferating cells, the increase of Gli1 signaling promotes cell proliferating but may also lose its inhibition to Caspase-3 or activates other signaling transduction pathways that increase the cell’s drug sensitivity.

Based on the results derived from this research, we propose a model for the novel function of the human oncogene Stil in the regulation of toxic susceptibility of DA or DA-like neurons. It functions through the Shh signal transduction pathway but the underlying mechanisms are different in mature and proliferating neurons (Fig. 4). In mature PC12 cells, the expression of Stil is required for cell survival in response to toxic drug treatment. Overexpression of Stil increases Shh signaling transduction and inhibits Caspase-3 activity, and thereby prevents cell death after 6-OHDA treatment. Shh-mediated inhibition of Caspase-3 activity has been reported in various cell types^28^,^29^,^30^,^31^. Down-regulation of Stil expression decreases Gli1 transcription, which then decreases its inhibition to Caspase-3 and thereby increases cell death in response to toxic drug treatment. In proliferating PC12 cells, down-regulation of Stil expression decreases the rate of cell proliferation but produces no effects on cell maturation or survival in response to neurotoxins. The increase of Stil expression leads to increases in drug susceptibility. This may be regulated by different mechanisms. One possibility is that excessive Shh signaling activates anti-Shh signaling pathways, which in turn, diminishes the inhibition of Gli1to Caspase-3^32^. Another possibility is that overexpression of Gli1 activates other yet unknown biochemical pathways that either directly or indirectly increase the cell’s drug sensitivity^33^,^34^. While the underlying mechanisms remain to be further examined, the data from this study provide evidence for the novel function of the human oncogene Stil in drug susceptibility in vertebrate neurons.

## Methods

### Cell culture and NGF induction

Proliferating PC12 cells were cultured in complete medium DMEM (4.5 g/L glucose, 110 mg/L sodium pyruvate, 25 mM HEPES; Hyclone) supplemented with 0.1% antibiotic-antimycotic solution (Fisher Scientific), 2 mM L-glutamine, 5% fetal bovine serum, and 10% donor horse serum (Hyclone). Medium was changed every 2 days, and cells were passaged every 3–5 days. For NGF induction, suspension PC12 cells in complete medium were seeded (20,000/cm^2^) onto culture plates coated with 10 μg/cm^2^ rat tail collagen (Sigma). After attachment (24 hours), medium was removed and replaced with 2% serum DMEM (0.1% antibiotic-antimycotic solution, 2 mM L-glutamine, 1% fetal bovine serum, and 1% donor horse serum) supplemented with 50 ng/mL 7 S NGF (Sigma). Medium with NGF was changed every other day. Maximal induction of neurite outgrowth in PC12 cells was observed after 4–6 days of NGF induction.

### Gene transfection

Adherent cells were cultured for 3–5 days (60–70% confluency) in complete medium, trypsinized, and then passed through a 22-gauge needle in order to obtain singular cells. Approximately 5 million cells and 10 μg DNA plasmid were used for each transfection using the Cell Line Nucleofector Kit V (Lonza). For shRNA transfections, cells were seeded for experiments after 72 hours. For overexpression transfections, cells were seeded for experiments after 24 hours. Control transfections (with scramble sequence) were performed along each experiment. In all cases, data obtained from the control experiments were normalized to 1.

### RT-PCR

Total RNA was extracted from PC12 cells using Trizol (Invitrogen) and reverse-transcribed by M-MLV reverse transcriptase (Promega). RT-PCR was performed according to standard protocols, and the melting curve analysis was performed using IQ5 software (Bio-Rad). Primer sequences: Stil, forward 5′-TATGGGCTTGCTGCTTGAGATAC-3′, reverse 5′-CAGG TTCCTTATGTGTCAATGAA-3′; Gli1, forward 5′-AGCTCCTGTGTAATTACGTTCAGTC-3′, reverse 5′- GGCTCTGACTAACTTGAGAACCTC-3′; Gapdh, forward 5′- GCACAGTCAA GGCCGAGAAT-3′, reverse 5′-GCCTTCTCCATGGTGGTGAA-3′.

### Western blot

Cells were lysed in freshly prepared lysis buffer (50 mM Tris-HCl pH 7.4; 150 mM NaCl; 1% nonidet 40; 5 mM DTT; 10 μg aprotinin; 10 μg leupeptin; 10 mM PMSF; 10% glycerol) on ice, and centrifuged at 12,000 rpm for 15 min. The supernatant was saved as whole-cell lysate, and protein concentrations were measured using the Pierce BCA Assay Kit (Thermo Scientific). For immunoblotting, 50–100 μg of protein was separated on a 5% stacking and 8% separating SDS-PAGE gel. Protein was transferred in Tris-glycine buffer with 20% methanol onto nitrocellulose membranes, and blocked with 5% non-fat milk in TBST, pH 7.5. After blocking, membranes were incubated overnight at 4 °C with primary antibodies (polyclonal rabbit anti-STIL, 1:200; Santa Cruz Biotechnology; polyclonal rabbit anti-ACTIN, 1:3,000, Sigma; polyclonal rabbit anti-cleaved Caspase-3, 1:3,000, Abcam) diluted in fresh blocking solution. The membranes were washed with TBST, and then incubated for 1 hour at room temperature with secondary antibodies (goat anti-rabbit IgG conjugated with HRP, 1:3000 for STIL, 1:30,000 for ACTIN) diluted in fresh blocking solution. The membranes were washed with TBST, and protein expression was detected using the Super-Signal West Pico Chemiluminescent Substrate (Thermo Scientific).

### Drug treatment

Cyclopamine (Sigma) was dissolved in ethanol at a concentration of 1 mM and stored in −80 °C. Dilutions of 10 or 20 μM were made in medium for treatment. Ethanol treatment (0.1%) was used as a vehicle control. Purmorphamine (Cayman Chemical) was dissolved in DMSO at a concentration of 1 mM and kept chilled prior to use. Dilutions of 5 or 10 μM were made in medium for treatment. DMSO treatment (0.01%) was used as a vehicle control. Neurotoxin (6-OHDA; Sigma) was dissolved in 0.15% ascorbic acid (stock concentration: 1 mM; stored in −80 °C) and applied to the culture medium at 0, 25, 50, 75, and 100 uM, respectively. In all cases, the duration of drug treatment was 24 hours. Drug treatment was applied to both undifferentiated proliferating cell and differentiated mature cells.

### Cell survival and apoptosis assays

The MTT reduction assay was used to examine PC12 cell viability. MTT (Sigma) was dissolved in DPBS at 5.5 mg/mL. Cells were seeded in triplicate for each test. Medium was removed and replaced with 0.5 mg/mL MTT in DPBS. Cells were incubated in MTT for 1 hour at 37 °C in 5% CO_2._ An equal volume of solvent (acidified isopropanol; 0.1 M HCl with 10% Triton X-100) was added to lyse the cells via trituration. Absorbance was read at 570 nm with background subtraction at 650 nm.

Caspase-3 activity was determined using a One-Step cellular caspase activity assay with DEVD-AMC (EMB Chemicals). After 24 hours of 6-OHDA treatment, cells were incubated for 1 h at 37 °C with 3X One-Step caspase assay buffer (150 mM HEPES, pH 7.4, 450 mM NaCl, 150 mM KCl, 30 mM MgCl2, 1.2 mM EGTA, 1.5% nonidet P40, 0.3% CHAPS, 30% sucrose) with 150 μM DEVD-AMC, 30 mM DTT and 3 mM phenylmethane sulphonyl fluoride. Caspase activity was measured using an FLx800 microplate fluorescence reader (excitation at 360 nm and emission at 460 nm).

The TUNEL assay was performed in according to the manufactory’s protocol (Life Technologies). At the end of drug treatment, cells were fixed and incubated with a methanol solution containing 0.3% H_2_O_2_ and then incubated with the TUNEL reaction mixture for 1 hour at 37 °C. Using a microscopy, TUNEL-positive nuclei were counted in 10 randomly selected fields per cover slip, and converted to percentage by comparing TUNEL-positive counts with the total cell nuclei counts.

## Additional Information

**How to cite this article**: Li, L. *et al.* A novel function of the human oncogene Stil: Regulation of PC12 cell toxic susceptibility through the Shh pathway. *Sci. Rep.* **5**, 16513; doi: 10.1038/srep16513 (2015).
